# The implication of calf circumference and grip strength in osteoporosis and bone mineral density among hemodialysis patients

**DOI:** 10.1007/s10157-022-02308-8

**Published:** 2022-12-27

**Authors:** Moe Ozawa, Nobuhito Hirawa, Tatsuya Haze, Aiko Haruna, Rina Kawano, Shiro Komiya, Yuki Ohki, Shota Suzuki, Yusuke Kobayashi, Akira Fujiwara, Sanae Saka, Masaaki Hanaoka, Hiroshi Mitsuhashi, Satoshi Yamaguchi, Toshimasa Ohnishi, Kouichi Tamura

**Affiliations:** 1 Department of Nephrology and Hypertension, Yokohama City University Medical Center, 4-57, Urafune-Cho, Minami-Ku, Yokohama, Japan; 2 Department of Medical Science and Cardiorenal Medicine, Yokohama City University Graduate School of Medicine, Yokohama, Japan; 3 YCU Center for Novel and Exploratory Clinical Trials (Y-NEXT), Yokohama City University Hospital, Yokohama, Japan; 4 Kamiooka Jinsei Clinic, Kousaikai Medical Corporation, Yokohama, Japan; 5 Yokohama Jinsei Hospital, Kousaikai Medical Corporation, Yokohama, Japan

**Keywords:** Hemodialysis, Osteoporosis, Sarcopenia

## Abstract

**Background:**

Chronic kidney disease–mineral and bone disorder (CKD–MBD), nutritional status, and uremia management have been emphasized for bone management in hemodialysis patients. Nevertheless, valuable data on the importance of muscle mass in bone management are limited, including whether conventional management alone can prevent osteoporosis. Thus, the importance of muscle mass and strength, independent of the conventional management in osteoporosis prevention among hemodialysis patients, was evaluated.

**Methods:**

Patients with a history of hemodialysis 6 months or longer were selected. We assessed the risk for osteoporosis associated with calf circumference or grip strength using multivariable adjustment for indices of CKD–MBD, nutrition, and dialysis adequacy. Moreover, the associations between bone mineral density (BMD), calf circumference, grip strength, and bone metabolic markers were also evaluated.

**Results:**

A total of 136 patients were included. The odds ratios (95% confidence interval) for osteoporosis at the femoral neck were 1.25 (1.04–1.54, *P* < 0.05) and 1.08 (1.00–1.18, *P* < 0.05) per 1 cm shorter calf circumference or 1 kg weaker grip strength, respectively. Shorter calf circumference was significantly associated with a lower BMD at the femoral neck and lumbar spine (*P* < 0.001). Weaker grip strength was also associated with lower BMD at the femoral neck (*P* < 0.01). Calf circumference or grip strength was negatively correlated with bone metabolic marker values.

**Conclusion:**

Shorter calf circumference or weaker grip strength was associated with osteoporosis risk and lower BMD among hemodialysis patients, independent of the conventional therapies.

**Supplementary Information:**

The online version contains supplementary material available at 10.1007/s10157-022-02308-8.

## Introduction

Osteoporosis is one of the major problems in the aging society, as it not only impairs the quality of life because of fractures but also increases the risk of mortality [1]. The frequency of femoral neck fractures is approximately fivefold to sixfold higher in dialysis patients than in healthy subjects [2]. In addition, hemodialysis patients with fractures have an approximately fourfold higher mortality risk [3]. Patients with chronic kidney disease (CKD) are afflicted with several bone metabolism-related conditions, one of which is the CKD-mineral and bone disorder (CKD–MBD) [4]. Typically, CKD causes secondary hyperparathyroidism with increased fibroblast growth factor 23, parathyroid hormone (PTH), and serum phosphorus (P), which affects bone metabolism. Moreover, uremic substances are detrimental to the bone and reduce elastic mechanical properties [5]. For these disease-specific conditions, the Kidney Disease Improving Global Organization and The European Renal Association–European Dialysis and Transplant Association have indicated the importance of controlling CKD–MBD, nutrition, and dialysis adequacy in the bone management of dialysis patients [6, 7].

On the other hand, sarcopenia, which is an equally important problem in an aging society, is exacerbated by CKD because of uremic toxins, oxidative stress, chronic inflammation, and malnutrition [8]. Approximately, 20% of dialysis patients have been reported to have sarcopenia [9]. Thus, hemodialysis patients have specific bone and muscle pathologies.

Bone mineral density (BMD) management is clinically important in osteoporosis and fracture prevention because osteoporosis is diagnosed through bone mass, and low BMD is a risk factor for fractures in hemodialysis patients [10]. Although previous studies have reported the association between low BMD and loss of appendicular skeletal muscle mass in the general population [11, 12], literature elucidating whether a similar relationship also applies to hemodialysis patients is limited. Furthermore, it is unclear whether managing CKD–MBD, nutrition, and uremia alone is sufficient to prevent osteoporosis in hemodialysis patients. In this study, we investigated whether the association between osteoporosis and muscle mass or strength was independent of conventional management to explore the importance of muscle mass and strength for preventing osteoporosis among hemodialysis patients.

## Materials and methods

### Study design and participants

This is a single-center, cross-sectional study. We screened patients aged over 20 years, who underwent hemodialysis three times a week at Kamiooka Jinsei Clinic (Yokohama, Japan) between July 2020 and April 2021. 200 individuals, who had a history of hemodialysis longer than 6 months and consented to this study, were selected. Among these 200 candidates, we excluded 64 patients (1) who had a history of lower-limb amputation, paralysis of limbs, use of steroids, or bone metastasis of cancer; (2) who were currently undergoing peritoneal dialysis; (3) who were currently receiving anti-osteoporosis drugs; or (4) whose record included a missing value required in the study. Finally, 136 patients were included in our analysis.

### Measurement

#### Bone mineral density

Mean BMD at each position of the lumbar spine (L2–4) and femoral neck was measured by dual-energy X-ray absorptiometry (DXA, Aria Chorale; GE Healthcare Japan Corporation, Tokyo, Japan). For measurement of the lumbar spine, vertebrae with focal changes (i.e., sclerotic changes) or artifacts were excluded. The mean BMD of two or more vertebrae, their T-score, and young adult mean (YAM) were evaluated. Osteoporosis was defined as T-score ≤  − 2.5 in accordance with the definition of the World Health Organization (WHO) [13].

#### Muscle mass and strength

We measured calf circumference and grip strength [14]. For calf circumference, the circumference of the thickest part of the lower leg was measured with a measuring tape, and the mean value of both sides was calculated. Grip strength was measured using a digital grip strength system (jammer type, MG-4800; MORITOH Co., Aichi, Japan), with the elbow joint bent at a 90° angle in the sitting position. The measurements were taken four times, alternating left and right twice, and the maximum value was obtained.

#### Nutritional indices

We calculated two nutritional indices, Nutritional Risk Index for Japanese Hemodialysis Patients (NRI-JH) and Geriatric Nutritional Risk Index (GNRI), to assess the nutritional state of each participant [15, 16].

#### Bone metabolic markers

We measured bone-specific alkaline phosphatase (BALP) and total type I procollagen N-terminal propeptide (P1NP) as bone formation markers and serum tartrate-resistant acid phosphatase 5b (TRACP-5b) as bone resorption marker [17].

#### Others

Blood samples were obtained on the first dialysis day of the week, 2 days after the previous dialysis day. The behavioral characteristics and clinical history were obtained using a questionnaire and medical records. For details of our methods, see Supplementary methods

### Statistical analyses

All analyses were performed by EZR on R Commander version 1.55. Significance was defined as *P* < 0.05 using two-sided tests.

First, we estimated the odds ratio (OR) for osteoporosis per 1 cm shorter calf circumference or 1 kg weaker grip strength using logistic regression models. Model 1 was unadjusted. Model 2 was adjusted for age, sex, history of diabetes, current smoking status, and habitual alcohol drinking. Model 3 was adjusted for the covariates included in Model 2 as well as serum hemoglobin (Hb), NRI-JH score, intact PTH (iPTH), and Kt/V. Covariates were selected a priori [18, 19]. Since we expected calf circumference and grip strength to be strongly correlated with BMI, we did not select BMI in our main models to avoid multicollinearity. Instead, we analyzed models that included height or DW as sensitivity analyses to ensure that the results did not change when taking body size into account. To confirm the linear relationship between calf circumference or grip strength and the risk for osteoporosis, we estimated adjusted ORs using the covariates in Model 3 between the tertiles based on calf circumference [i.e., Low ≤ 33.4, 33.5 ≤ Medium ≤ 36.5, 36.6 ≤ High (cm)] or grip strength [i.e., Low ≤ 22.3, 22.8 ≤ Medium ≤ 30.9, 31.2 ≤ High (cm)] using the highest group as a reference.

Second, we estimated the Pearson’s product–moment correlation coefficients and 95% confidence interval (CI) for the correlation between BMD and calf circumference or grip strength.

Third, we used multiple linear regression models to calculate standardized β and 95% CI for the association of BMD with calf circumference or grip strength. The linear models were adjusted for the same covariates as in the logistic regression models.

Fourth, we assessed the association between bone metabolic markers and calf circumference, grip strength, or BMD by scatter plotting and correlation tests.

### Sensitivity analyses

We performed the following sensitivity analyses by modifying some of the covariates included in Model 3: (1) additionally adjusted for height or dry weight when assessing calf circumference or grip strength, respectively; (2) used GNRI score instead of NRI-JH score; (3) used serum albumin (Alb) instead of NRI-JH score; (4) used corrected serum calcium (Ca) and P levels instead of iPTH; (5) used CKD–MBD drug (i.e., vitamin D receptor activators, phosphate binders, or calcimimetics) instead of iPTH; (6) used log-transformed hemodialysis duration instead of Kt/V.

### Subgroup analyses by iPTH or sex

To check the heterogeneity in the association between BMD and calf circumference or grip strength by the levels of iPTH, we performed the subgroup analysis between the low- and high-iPTH groups (i.e., iPTH < 147.5 pg/mL, iPTH ≥ 147.5 pg/mL according to the median value) and included multiplicative interaction terms in the regression models. We also performed subgroup analyses of the female and male groups.

## Results

### Clinical characteristics

The final analytic sample included 136 patients. Table 1 shows the baseline characteristics. The mean ± standard deviation (SD) of age was 67.4 ± 12.7 years and 25.0% of the subjects were female. The levels of BMD at the femoral neck and lumbar spine were 0.8 ± 0.1 g/cm^2^ and 1.2 ± 0.3 g/cm^2^, respectively. The mean calf circumference was 35.3 ± 4.0 cm and the grip strength was 26.9 ± 9.7 kg.

**Table 1:** Characteristics of participants

	Overall group (*n* = 136)
Clinical and behavioral characteristics	
Age, years	67.4 ± 12.7
Female, *n* (%)	34 (25.0)
Height, m	1.6 ± 0.1
Dry weight, kg	62.6 ± 14.2
BMI *, kg/m^2^	23.1 ± 4.4
Current smoking, *n* (%)	16 (11.8)
Habitual drinking, *n* (%)	38 (27.9)
Hemodialysis condition	
Hemodialysis duration, years	7.5 (3.3, 11.6)
Kt/V	1.4 ± 0.3
Laboratory data	
Hemoglobin, g/L	111.1 ± 10.8
Serum albumin, g/L	37.2 ± 3.0
Corrected serum calcium †, mmol/L	2.3 ± 0.2
Serum phosphorus, mmol/L	1.7 ± 0.4
IPTH, pg/mL	156.8 ± 84.3
Cause of end-stage renal disease	
Glomerulonephritis, *n* (%)	45 (33.1)
Diabetic nephropathy, *n* (%)	58 (42.6)
Nephrosclerosis, *n* (%)	15 (11.0)
Polycystic kidney disease, *n* (%)	8 (5.9)
Unknown or others, *n* (%)	10 (7.4)
History of complications	
Diabetes, *n* (%)	63 (46.3)
Parathyroidectomy, *n* (%)	5 (3.7)
CVD event ‡, *n* (%)	47 (34.6)
Medications	
Vitamin D receptor activators, *n* (%)	101 (74.3)
Phosphate binders, *n* (%)	119 (87.5)
Calcimimetics, *n* (%)	49 (36.0)
Bone mass	
Femoral neck	
BMD, g/cm^2^	0.8 ± 0.1
T-score	− 1.9 ± 1.1
YAM, %	77.3 ± 14.0
Lumbar spine	
BMD, g/cm^2^	1.2 ± 0.3
T-score	0.3 ± 1.7
YAM, %	104.9 ± 23.3
Measurements of muscle mass and strength	
Calf circumference, cm	35.3 ± 4.0
Grip strength, kg	26.9 ± 9.7
Nutritional indices	
NRI-JH score	3.1 ± 3.0
GNRI score	104.8 ± 10.7

Values for continuous variables are expressed as mean ± standard deviation for unskewed variables and median (25th and 75th percentiles) for skewed variables. Values for categorical variables are expressed as percentages. BMD was calculated using dual-energy X-ray absorptiometry. T-score was calculated as the standard deviation from the mean BMD of Japanese young adults aged 20–29 years for the femoral neck and 20–44 years for the lumbar spine. YAM was calculated as a percentage of the subject’s BMD relative to the average BMD value of Japanese young adults

*Calculated as follows: dry weight / (height, m)^2^

†Corrected as follows (expressed in mmol/L): calcium (g/dL) + [4.0 − serum albumin (g/dL)], if albumin < 4.0 g/dL

‡Defined as angina requiring cardiac catheterization, myocardial infarction, or stroke

*BMD* bone mineral density, *BMI* body mass index, *CVD* cardiovascular disease, *DM* diabetes mellitus, *GNRI* Geriatric Nutritional Risk Index, *iPTH* intact parathyroid hormone, *NRI-JH* Nutritional Risk Index for Japanese Hemodialysis Patients, *YAM* young adult mean

### Risk for osteoporosis among hemodialysis patients with decreased calf　circumference or grip strength

**Fig. 1:**
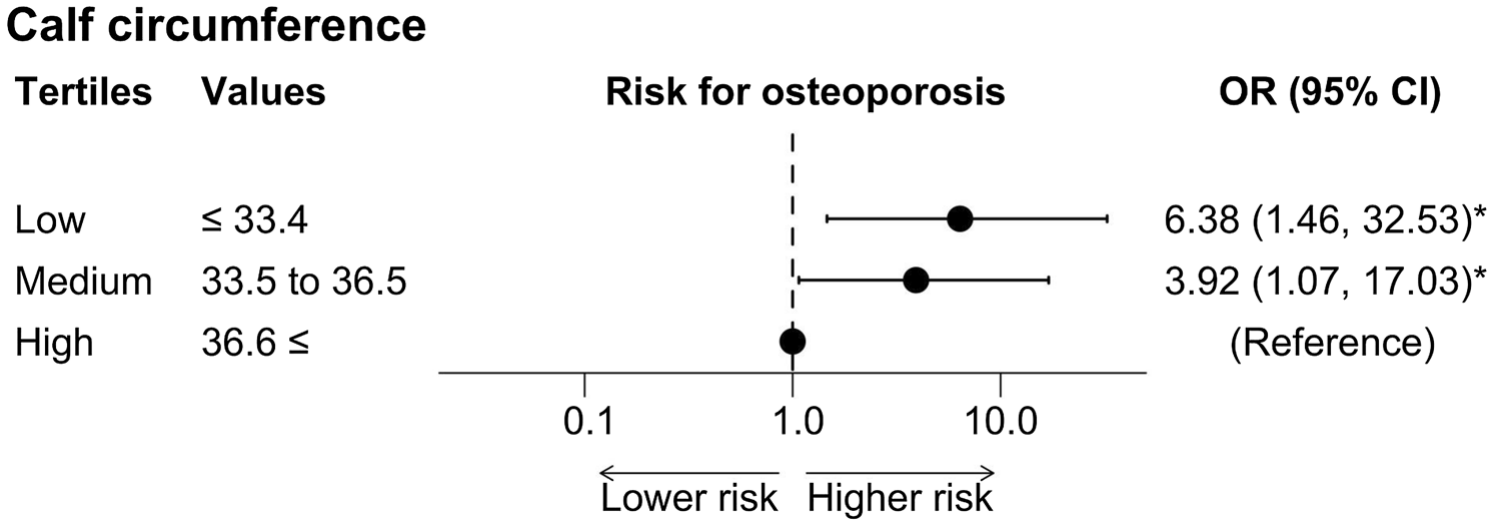
Risk for osteoporosis in tertiles based on calf circumference. Adjusted estimated odds ratios (95% confidence intervals) for osteoporosis in tertiles based on calf circumference [i.e., Low ≤ 33.4 vs. 33.5 ≤ Medium ≤ 36.5 vs. 36.6 ≤ High (cm)] are shown. The estimated odds ratios were adjusted for age, sex, history of diabetes, current smoking status, habitual alcohol drinking, hemoglobin, NRI-JH score, iPTH, and Kt/V. iPTH = intact parathyroid hormone; NRI-JH = nutritional risk index for Japanese hemodialysis patients. *P < 0.05

Forty-five patients, consisting of 23/34 (67.6%) women and 22/102 (21.6%) men, were diagnosed with osteoporosis at the femoral neck. At the lumbar spine, six patients (all women) were diagnosed with osteoporosis. As shown in Table 2, when the risk for osteoporosis diagnosed at the femoral neck was assessed, the estimated OR (95% CI) for osteoporosis per 1 cm shorter calf circumference was 1.25 (1.04–1.54, *P* < 0.05). The estimated OR for osteoporosis per 1 kg weaker grip strength was 1.08 (1.00–1.18, *P* < 0.05). In the analysis with calf circumference as a variable (model 3), being female was a significant risk factor for osteoporosis. Furthermore, when calf circumference was divided into three groups according to the tertiles in Fig. 1, the shortest group (Low) and the intermediate group (Medium) were at significantly higher risk for osteoporosis compared to the longest group (High) [OR 6.38 (1.46–32.53), *P* < 0.05**;** OR 3.92 (1.07–17.03), *P* < 0.05, respectively]. For grip strength, although not significant, the Low and Medium groups showed higher ORs compared to the High group in terms of the point estimates [OR 4.80 (0.96–28.60), *P* = 0.06; OR 3.70 (0.96–18.38), *P* = 0.06, respectively]. No evidence was found to suggest that there was a non-linear relationship between decreased calf circumference or grip strength and the risk for osteoporosis.

**Table 2:** Risk for osteoporosis diagnosed at the femoral neck with decreased calf circumference or grip strength

Odds ratio for osteoporosis		
	Calf circumference per 1 cm shorter	Grip strength per 1 kg weaker
Model 1 (unadjusted)	1.36 (1.20, 1.57) **	1.14 (1.09, 1.21) **
Model 2	1.22 (1.03, 1.46) *	1.08 (1.01, 1.17) *
Model 3	1.25 (1.04, 1.54) *	1.08 (1.00, 1.18) *

Unadjusted and adjusted odds ratios (95% confidence interval) for osteoporosis associated with one-unit decrement of calf circumference or grip strength are shown. Osteoporosis was defined as T-score ≤ − 2.5 at the femoral neck. Model 1 was unadjusted. Model 2 was adjusted for age, sex, history of diabetes, current smoking status, and habitual alcohol drinking. Model 3 was adjusted for the covariates included in Model 2 as well as hemoglobin, NRI-JH score, iPTH, and Kt/V. Exposures were included in models separately

*P < 0.05; **P < 0.001

*iPTH* intact parathyroid hormone, *NRI-JH* Nutritional Risk Index for Japanese Hemodialysis Patients

### Association between BMD and muscle mass and strength

**Fig. 2:**
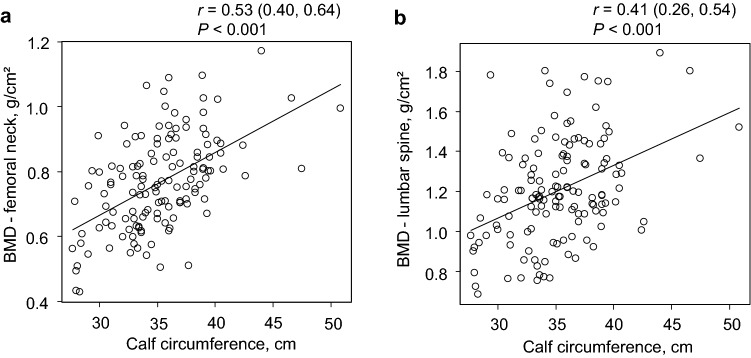
Scatter plots of the relationships between BMD and calf circumference. Scatter plots of the relationships between BMD at the **a** femoral neck or **b** lumbar spine and calf circumference among hemodialysis patients are shown. Each circle represents an individual value. The black lines represent simple linear regression models. The *P* values were calculated for Pearson’s product–moment correlation coefficients (*r*-values). *BMD* bone mineral density

**Fig. 3:**
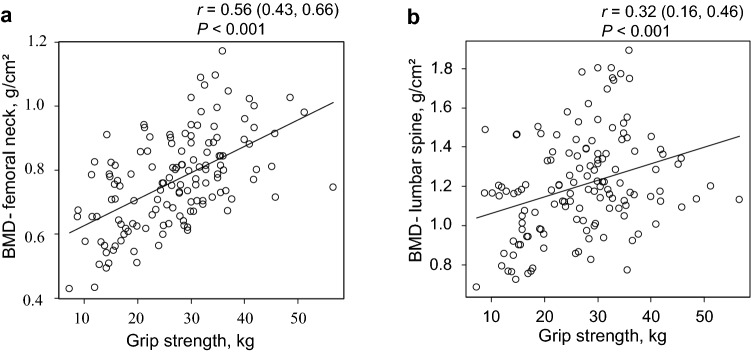
Scatter plots of the relationships between BMD and grip strength. Scatter plots of the relationships between BMD at the **a** femoral neck or **b** lumbar spine and grip strength among hemodialysis patients are shown. Each circle represents an individual value. The black lines represent simple linear regression models. The *P*-values were calculated for Pearson’s product–moment correlation coefficients (*r*-values). BMD = bone mineral density

As shown in Fig. 2, calf circumference was significantly correlated with BMD at both the femoral neck [*r* (95% CI) = 0.53 (0.40–0.64), *P* < 0.001] and the lumbar spine [0.41 (0.26–0.54), *P* < 0.001]. Similarly, grip strength showed correlations with BMD at both the femoral neck [0.56 (0.43–0.66), *P* < 0.001] and the lumbar spine [0.32 (0.16–0.46), *P* < 0.001] (Fig. 3).

In the unadjusted model of linear regression analyses (Table 3), calf circumference and grip strength were associated with BMD at both the femoral neck and lumbar spine. After multivariable adjustment including the indices for managing CKD–MBD, nutrition, and dialysis adequacy (i.e., iPTH, NRI-JH score, and Kt/V), shorter calf circumference was significantly associated with lower BMD at both the femoral neck [β (95% CI) = 0.36 (0.18–0.55), *P* < 0.001] and the lumbar spine [0.37 (0.16 to 0.56), *P* < 0.001]. Weaker grip strength was also associated with lower BMD at the femoral neck [0.32 (0.11–0.53), *P* < 0.01]. Women, non-DM patients, and older age showed significant differences in the association with BMD in our models, while iPTH, NRI-JH score, and Kt/V did not.

**Table 3:** Association between BMD and calf circumference or grip strength among hemodialysis patients

Standardized b		
	Calf circumference	Grip strength
Femoral neck		
Model 1 (unadjusted)	0.53 (0.38, 0.67) **	0.55 (0.41, 0.70) **
Model 2	0.37 (0.20, 0.54) **	0.36 (0.16, 0.56) **
Model 3	0.36 (0.18, 0.55) **	0.32 (0.11, 0.53) *
Lumbar spine		
Model 1 (unadjusted)	0.41 (0.25, 0.56) **	0.32 (0.16, 0.48) **
Model 2	0.36 (0.18, 0.54) **	0.15 (-0.07, 0.36)
Model 3	0.37 (0.17, 0.56) **	0.09 (-0.14, 0.32)

Unadjusted and adjusted standardized b values (95% confidence interval) for BMD associated with calf circumference or grip strength are shown. Model 1 was unadjusted. Model 2 was adjusted for age, sex, history of diabetes, current smoking status, and habitual alcohol drinking. Model 3 was adjusted for the covariates included in Model 2 as well as hemoglobin, NRI-JH score, iPTH, and Kt/V. Exposures were included in the models separately

*P < 0.01; **P < 0.001

*BMD* bone mineral density, *iPTH* intact parathyroid hormone, *NRI-JH* Nutritional Risk Index for Japanese Hemodialysis Patients

### Sensitivity analyses and subgroup analyses

We conducted six sensitivity analyses including body size correction, other nutritional indices, and CKD–MBD drug, the results of which were similar in terms of the point estimates (Supplementary Tables S1 and S2).

Furthermore, in the subgroup analysis by iPTH levels, calf circumference was significantly associated with BMD in both groups (**Supplementary Table S3**). No evidence was found to suggest that iPTH levels interacted in the association between BMD and calf circumference or grip strength (all *P* for interaction > 0.61). Subgroup analysis by sex also showed a significant association between calf circumference and BMD in both groups (Supplementary Table S4).

### Correlations between bone metabolic markers and calf circumference or grip strength

**Fig. 4:**
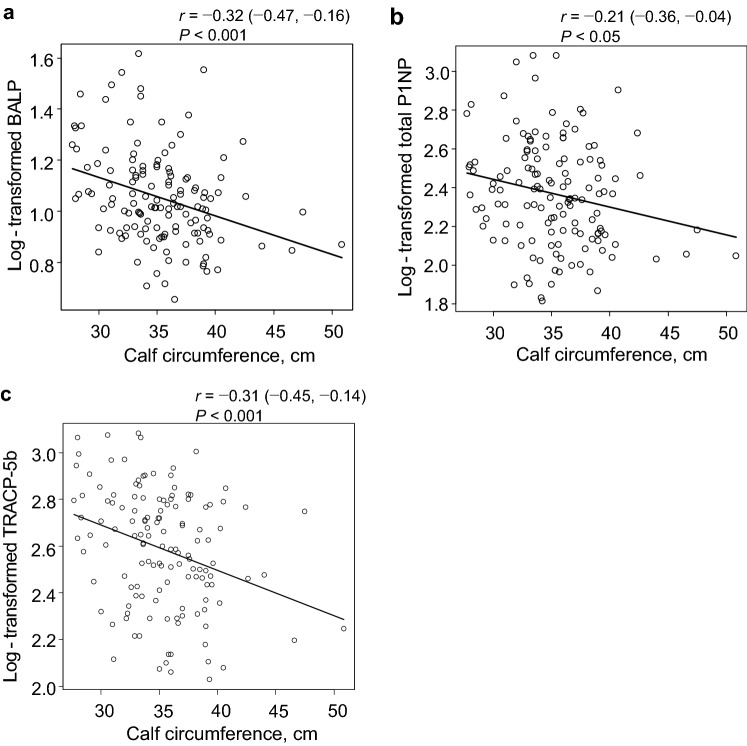
Scatter plots of the relationships between bone metabolic markers and calf circumference. Scatter plots of the relationships between log-transformed **a** BALP, **b** total P1NP, or **c** TRACP-5b and calf circumference are shown. Each circle represents an individual value. The black lines represent simple linear regression models. The *P* values were calculated for Pearson’s product–moment correlation coefficients (*r*-values). BALP = bone-specific alkaline phosphatase; P1NP = type I procollagen N-terminal propeptide; TRACP-5b = tartrate-resistant acid phosphatase 5b

The scatter plots of bone metabolic markers and calf circumference or grip strength are shown in Fig. 4 and Supplementary Fig. S1, respectively. Calf circumference was negatively correlated with log-transformed BALP [*r* (95% CI) =  − 0.32 (− 0.47 to − 0.16), *P* < 0.001], total P1NP [− 0.21 (− 0.36 to − 0.04), *P* < 0.05], or TRACP-5b [− 0.31 (− 0.45 to − 0.14), *P* < 0.001]. Grip strength was also inversely correlated with log-transformed BALP [− 0.33 (− 0.47 to − 0.17), *P* < 0.001], total P1NP [− 0.28 (− 0.43 to − 0.12), *P* < 0.001], or TRACP-5b [− 0.33 (− 0.48 to − 0.18), *P* < 0.001]. Furthermore, in subgroup analysis using the median iPTH as a cutoff, calf circumference was negatively correlated with log-transformed BALP and TRACP5b in both subgroups (Supplementary Table S5). These metabolic markers were also inversely correlated with BMD (Supplementary Fig. S2).

## Discussion

In the general Japanese population, the prevalence of osteoporosis diagnosed at the femoral neck is reported to be 22.2% in women and 7.0% in men aged 60–69 years [20]. In this study of hemodialysis patients, osteoporosis diagnosed was approximately three times higher than in the general population. Furthermore, in hemodialysis patients, calf circumference and grip strength were significantly associated with the risk for osteoporosis. These associations were independent of the nutritional state (i.e., NRI-JH, GNRI, and serum Alb), CKD–MBD indices (i.e., serum Ca, serum P, iPTH, and use of vitamin D, phosphate binders, or calcimimetics), and dialysis adequacy (i.e., Kt/V). Calf circumference is related to appendicular skeletal muscle mass, and calf circumference and grip strength are used as surrogate markers for the diagnosis of sarcopenia [14, 21]. Hence, our findings may suggest a close association between sarcopenia and osteoporosis in hemodialysis patients.

Similarly, in a multivariate analysis, shorter calf circumference or weaker grip strength were also associated with low BMD. We assessed the heterogeneity in these associations between sexes [22], and the association between muscle mass and BMD was significant in both sexes. We also demonstrated that calf circumference and BMD were inversely correlated with both bone formation and resorption markers. In this study, lower muscle mass and BMD were associated with a higher bone turnover as elevations in both bone formation and resorption markers indicate increased bone metabolism [23]. Because iPTH promotes bone resorption and affects bone rotation [4], this association was also examined in the subgroup analysis of iPTH. The results showed that calf circumference was significantly correlated with BALP or TRACP-5b in both groups. Several reports have already shown that the elevation of these resorption and formation markers was strongly associated with a rapid decrease in BMD and risk for fractures [24]. Thus, our findings indicate that the loss of muscle mass may be associated with a rapid BMD decline due to a high bone turnover and thus require early intervention.

Recently, it has been clarified that muscle can endocrinologically affect bone metabolism via myokines. Myostatin, which promotes bone resorption, is negatively correlated with muscle mass, and elevated myostatin levels may be related to lower BMD in sarcopenia [25]. Additionally, irisin, whose secretion is increased through exercise, inhibits bone resorption via the suppression of receptor activator of nuclear factor κB ligand expression [26]. Therefore, maintaining muscle mass may suppress bone resorption and preserve BMD through these myokines.

In a cross-sectional study of hemodialysis patients, Tominaga et al. and Ito et al. showed that grip strength was not associated with BMD, whereas muscle mass was significantly associated with BMD [21, 27]. In a study of 131 patients undergoing hemodialysis, Lee et al. reported that upper arm circumference and skeletal muscle mass index were lower in the osteopenia and osteoporosis groups than in the normal group [28]. Although these previous studies reported the association between low muscle mass or strength and low BMD or osteoporosis in hemodialysis patients, the association has not been adequately investigated, adjusting for factors (CKD–MBD, nutrition, and uremia) that have traditionally been considered risk factors for osteoporosis. Our results clearly demonstrated this association in hemodialysis patients and indicate the possibility that maintenance or appendicular muscle training may be needed to prevent osteoporosis, in addition to the conventional management of CKD–MBD, nutrition, and dialysis adequacy. Furthermore, we found that lower muscle mass was associated with higher levels of bone metabolic markers and lower BMD in hemodialysis patients. Because dialysis patients are sometimes unable to perform sufficient exercise because of their complications (e.g., heart failure and cerebral infarction), they will have an increased risk of a low BMD. Therefore, a bone resorption inhibitor may be useful for patients who have difficulty performing proper exercise.

In our multiple regression models, the association between grip strength and BMD at the lumbar spine did not reach statistical significance. Bone mass at the lumbar spine of hemodialysis patients was high, despite the exclusion of spines with localized changes on imaging findings. Aortic calcification, a common complication among dialysis patients, affect BMD values at the lumbar spine assessed using DXA [29]. Thus, the femoral neck may be more suitable than the lumbar spine in the assessment of BMD in hemodialysis patients. Also, older age, women, and non-DM patients were significantly associated with osteoporosis risk or lower BMD in our multivariate model. Aging and women are known risk factors for osteoporosis and lower BMD [30], consistent with our results. It has been reported that osteocalcin levels, a marker of bone formation, are decreased in DM patients, and that osteocalcin increases as blood glucose improves [31]. On the other hand, large studies reported that DM patients have rather higher BMD than non-DM patients [32, 33]. The mechanism by which BMD is higher in patients with DM is still unknown; however, it is hypothesized that insulin promotes bone formation by interacting with IGF-1 receptor [34]. In the present study, decreased calf circumference or grip strength was associated with the risk for osteoporosis or lower BMD, even when adjusted for the presence of DM.

This study has several limitations. First, we could not measure muscle mass directly, which might be helpful for the precise diagnosis of sarcopenia. Second, the causal relationship between muscle mass and BMD could not be revealed because of the observational nature of this study. However, it is reasonable to assume that exercise therapy may improve BMD in dialysis patients because it has already been shown that adequate exercise increases BMD in healthy subjects [35]. Prospective interventional studies are needed to confirm this in the future.

## Conclusion

Shorter calf circumference and weaker grip strength were associated with osteoporosis risk and lower BMD in hemodialysis patients, independent of the management of CKD–MBD, nutrition status, and dialysis adequacy. In addition, BMD decline with loss of muscle mass may require earlier intervention due to higher bone turnover. To prevent osteoporosis in hemodialysis patients, clinicians should pay attention to calf circumference and grip strength, in addition to the conventional management.

## Supplementary Information

Below is the link to the electronic supplementary material.

Supplementary file1 (PDF 654 KB)

## Data Availability

The datasets generated and/or analyzed during the current study are available from the corresponding author on reasonable request.
