# Supplementary material for: The implication of calf circumference and grip strength in osteoporosis and bone mineral density among hemodialysis patients
Source: Clin Exp Nephrol. 2022 Dec 27;27(4):365–73. doi: 10.1007/s10157-022-02308-8 (PMC10023647; doi:10.1007/s10157-022-02308-8)
Supplement: Supplementary file 1 — Supplementary file1 (PDF 654 KB) [file 10157_2022_2308_MOESM1_ESM.pdf]

*Supplementary Material*

**The implication of calf circumference and grip strength in osteoporosis and bone mineral density among hemodialysis patients**


**Authors**

Moe Ozawa, MD[1,2)]; Nobuhito Hirawa, MD, PhD[1)]; Tatsuya Haze, MD, PhD[1-3)]; Aiko Haruna, MD[1,2)]; Rina Kawano, MD[1,2)]; Shiro Komiya, MD[1,2)]; Yuki Ohki, MD, PhD[1)]; Shota Suzuki, MD, PhD[1)]; Yusuke Kobayashi, MD, PhD[3)]; Akira Fujiwara, MD, PhD[1)]; Sanae Saka, MD, PhD[1)]; Masaaki Hanaoka, MD[4)]; Hiroshi Mitsuhashi, MD[4)]; Satoshi Yamaguchi, MD, PhD[5)]; Toshimasa Ohnishi, MD, PhD[4)]; and Kouichi Tamura, MD, PhD[2)]

**Affiliations**

1. Department of Nephrology and Hypertension, Yokohama City University Medical Center, Yokohama, Japan

2. Department of Medical Science and Cardiorenal Medicine, Yokohama City University Graduate School of Medicine, Yokohama, Japan

3. YCU Center for Novel and Exploratory Clinical Trials (Y-NEXT), Yokohama City University Hospital, Yokohama, Japan.

4. Kamiooka Jinsei Clinic, Kousaikai Medical Corporation, Yokohama, Japan

5. Yokohama Jinsei Hospital, Kousaikai Medical Corporation, Yokohama, Japan

**Correspondence to:**

**Nobuhito Hirawa, MD, PhD, FACP, FAHA, FJSIM, FJSH**

Department of Nephrology and Hypertension, Yokohama City University Medical Center

Address: 4-57, Urafune-cho, Minami-ku, Yokohama 232-0024, Japan.

TEL: +81-45-261-5656, FAX: +81-45-253-9955

E-mail: hirawa@yokohama-cu.ac.jp

# TABLE OF CONTENTS

**Supplementary Methods**

**Supplementary Table S1.** Risk for osteoporosis diagnosed at the femoral neck with decreased calf circumference or grip strength (sensitivity analysis)

**Supplementary Table S2.** Association between BMD and calf circumference or grip strength among hemodialysis patients (sensitivity analysis)

**Supplementary Table S3.** Association between BMD and calf circumference or grip strength among hemodialysis patients based on the subgroup analysis using the median iPTH as a cut-off

**Supplementary Table S4.** Association between BMD and calf circumference or grip strength among hemodialysis patients based on the subgroup analysis by sex

**Supplementary Table S5.** Correlations between bone metabolic markers and calf circumference or grip strength on the subgroup analysis using the median iPTH as a cut-off

**Supplementary Fig. S1.** Scatter plots of the relationships between bone metabolic markers and grip strength

**Supplementary Fig. S2.** Scatter plots of the relationships between BMD and bone metabolic markers

**Supplementary References**

# Supplementary Methods

*Bone mineral density*

T-score was calculated as the standard deviation (SD) from the mean BMD of Japanese young adults, aged 20–29 years for the femoral neck and 20–44 years for the lumbar spine. Young adult mean was calculated as the percentage of each subject's BMD relative to the mean BMD value of Japanese young adults. In principle, BMD at the femoral neck was measured with the left leg unless there were artifacts caused by artificial bone head or metal fixation.

*Bone metabolism markers*

IPTH and total P1NP were measured by electrochemiluminescence immunoassay (ECLIA). BALP was measured by chemiluminescent enzyme immunoassay (CLEIA). TRACP-5b was measured by enzyme immunoassay (EIA). Levels over the detection

limit for total P1NP were considered equivalent to 1201 μg/L in this study because the detection limit was 1200 μg/L (n = 2).

*Nutritional indices*

Nutritional Risk Index for Japanese Hemodialysis Patients (NRI-JH) is known to be an index of malnutrition, associated with 1-year mortality risk in hemodialysis patients [1]. NRI-JH was scored using post-dialysis body mass index (BMI), pre-dialysis serum albumin (Alb), pre-dialysis serum total cholesterol (TC), and pre-dialysis serum creatinine (Cre) as follows:

low BMI (BMI < 20 kg/m$^2$), yes = 3, no = 0;

low Alb (Alb < 3.7 g/dL when age < 65, < 3.5 g/dL when age ≥ 65), yes = 4, no = 0;

low TC (TC < 130 mg/dL), yes = 1, no = 0;

high TC (TC ≥ 220 mg/dL), yes = 2, no = 0;

low Cre (Cre < 9.7 mg/dL for females aged < 65, Cre < 8.0 mg/dL for females aged ≥ 65, Cre < 11.6 mg/dL for males aged < 65,

Cre < 9.7 mg/dL for males aged ≥ 65), yes = 4, no = 0.

In this study, post-dialysis BMI was calculated by dividing dry weight (DW) by the squared height in meters.

Geriatric Nutritional Risk Index (GNRI) is also known as an index of malnutrition, which was scored based on serum Alb, current body weight, and ideal body weight using the following equation [2]:

GNRI = [1.489 × Alb (g/L)] + [41.7 × (measured BW / ideal BW)]

When measured BW ≥ ideal BW, (measured BW / ideal BW) was set to 1. In this study, ideal BW was calculated by the square of the height in meters, multiplied by 22 [3]. The DW was used for the measured BW.

*Others*

Serum calcium (Ca) concentration (expressed in mmol/L) was corrected by serum Alb as follows: Ca g/dL+ [(4.0 – serum Alb (g/dL)], if Alb < 4.0 g/dL.

DW was determined by each physician depending on the clinical findings. BMI was calculated by dividing DW by height squared.

We assessed the adequacy of hemodialysis by calculating Kt/V using dialysis time, blood urea nitrogen (BUN), and BW before and after dialysis on the first dialysis day of the week as follows: Kt/V = −Ln (post-dialysis BUN / pre-dialysis BUN − 0.008 × dialysis time) + (4 − 3.5 × post-dialysis BUN / pre-dialysis BUN) × weight loss / post-dialysis BUN [4, 5].

Diabetes mellitus was diagnosed in accordance with the guidelines of the Japan Diabetes Society [6].

**Supplementary Table S1. Risk for osteoporosis diagnosed at the femoral neck with decreased calf circumference or grip strength (sensitivity analysis)**

| Odds ratio for osteoporosis | | | |
|---|---|---|---|
| | | **Calf circumference per 1 cm shorter** | **Grip strength per 1 kg weaker** |
| Model 3 | Same as Model 3 in Table 2 | 1.25 (1.04, 1.54) * | 1.08 (1.00, 1.18) * |
| Model 4 | Adjusted for height or dry weight | 1.24 (1.02, 1.52) * | 1.08 (1.00, 1.17) |
| Model 5 | Adjusted for GNRI | 1.30 (1.05, 1.64) * | 1.07 (1.00, 1.16) |
| Model 6 | Adjusted for serum Alb | 1.22 (1.02, 1.48) * | 1.10 (1.01, 1.19) * |
| Model 7 | Adjusted for corrected serum calcium and phosphate | 1.25 (1.03, 1.54) * | 1.09 (1.00, 1.18) * |
| Model 8 | Adjusted for CKD-MBD drug use | 1.27 (1.04, 1.57) * | 1.10 (1.02, 1.21) * |
| Model 9 | Adjusted for log-transformed hemodialysis duration | 1.28 (1.06, 1.57) * | 1.09 (1.01, 1.19) * |

Adjusted odds ratios for osteoporosis (95% confidence interval) diagnosed based on T-score ≤ −2.5 at the femoral neck associated with one-unit decrement of calf circumference or grip strength are shown. Model 3 was the same model in Table 2 and was adjusted for age, sex, history of diabetes, current smoking status, habitual alcohol drinking, hemoglobin, NRI-JH score, iPTH, and Kt/V. Model 4 was adjusted for height or dry weight when using calf circumference or grip strength, respectively. Model 5 was adjusted for GNRI score instead of NRI-JH score. Model 6 was adjusted for serum Alb instead of NRI-JH score. Model 7 was adjusted for corrected serum calcium and phosphate instead of iPTH. Model 8 was adjusted for medication including the use of vitamin D receptor activators, phosphate binders, or calcimimetics instead of iPTH. Model 9 was adjusted for log-transformed hemodialysis duration instead of Kt/V. Alb = albumin; CKD-MBD = chronic kidney disease-mineral and bone disorder; GNRI = Geriatric Nutritional Risk Index; iPTH = intact parathyroid hormone; NRI-JH = Nutritional Risk Index for Japanese Hemodialysis Patients. *$P < 0.05$.

**Supplementary Table S2. Association between BMD and calf circumference or grip strength among hemodialysis patients (sensitivity analysis)**

| | | Standardized β | |
|---|---|---|---|
| | | **Calf circumference** | **Grip strength** |
| **Femoral neck** | | | |
| Model 3 | Same as Model 3 in Table 3 | 0.36 (0.18, 0.55) *** | 0.32 (0.11, 0.53) ** |
| Model 4 | Adjusted for height or dry weight | 0.34 (0.15, 0.52) *** | 0.27 (0.07, 0.47) ** |
| Model 5 | Adjusted for GNRI | 0.32 (0.11, 0.54) ** | 0.29 (0.09, 0.49) ** |
| Model 6 | Adjusted for serum Alb | 0.34 (0.16, 0.52) *** | 0.34 (0.13, 0.54) ** |
| Model 7 | Adjusted for corrected serum calcium and phosphate | 0.36 (0.18, 0.55) *** | 0.34 (0.13, 0.54) ** |
| Model 8 | Adjusted for CKD-MBD drug use | 0.37 (0.18, 0.55) *** | 0.36 (0.15, 0.57) *** |
| Model 9 | Adjusted for log-transformed hemodialysis duration | 0.39 (0.21, 0.58) *** | 0.37 (0.16, 0.57) *** |
| **Lumbar spine** | | | |
| Model 3 | Same as Model 3 in Table 3 | 0.37 (0.17, 0.56) *** | 0.09 (-0.14, 0.32) |

| | | | |
|---|---|---|---|
| Model 4 | Adjusted for height or dry weight | 0.36 (0.16, 0.56) *** | 0.02 (-0.19, 0.24) |
| Model 5 | Adjusted for GNRI | 0.25 (0.02, 0.48) * | 0.06 (-0.16, 0.28) |
| Model 6 | Adjusted for serum Alb | 0.35 (0.16, 0.53) *** | 0.13 (-0.10, 0.35) |
| Model 7 | Adjusted for corrected serum calcium and phosphate | 0.37 (0.17, 0.56) *** | 0.14 (-0.09, 0.36) |
| Model 8 | Adjusted for CKD-MBD drug use | 0.37 (0.17, 0.57) *** | 0.10 (-0.14, 0.33) |
| Model 9 | Adjusted for log-transformed hemodialysis duration | 0.39 (0.19, 0.58) *** | 0.13 (−0.10, 0.36) |

Adjusted standardized β values (95% confidence interval) for BMD associated with calf circumference or grip strength are shown. Model 3 was the same model in Table 3 and was adjusted for age, sex, history of diabetes, current smoking status, habitual alcohol drinking, hemoglobin, NRI-JH score, iPTH, and Kt/V. Model 4 was adjusted for height or dry weight when using calf circumference or grip strength, respectively. Model 5 was adjusted for GNRI score instead of NRI-JH score. Model 6 was adjusted for serum Alb instead of NRI-JH score. Model 7 was adjusted for corrected serum calcium and phosphate instead of iPTH. Model 8 was adjusted for medication including the use of vitamin D receptor activators, phosphate binders or calcimimetics instead of iPTH. Model 9 was adjusted for log-transformed hemodialysis duration instead of Kt/V. Alb = albumin; BMD = bone mineral density; CKD-MBD = chronic kidney disease-mineral and bone disorder; GNRI = Geriatric Nutritional Risk Index; iPTH = intact parathyroid hormone; NRI-JH = Nutritional Risk Index for Japanese Hemodialysis Patients. **P* < 0.05; ***P* < 0.01; ****P* < 0.001.

**Supplementary Table S3. Association between BMD and calf circumference or grip strength among hemodialysis patients based on the subgroup analysis using the median iPTH as a cut-off**

| | **Low-iPTH group (*n* = 68)** | | **High-iPTH group (*n* = 68)** | |
|---|---|---|---|---|
| | **Model 1 (Unadjusted)** | **Model 2 (Adjusted)** | **Model 1 (Unadjusted)** | **Model 2 (Adjusted)** |
| **Femoral neck** | | | | |
| **Calf circumference** | 0.52 (0.31, 0.73) *** | 0.45 (0.19, 0.71) *** | 0.55 (0.34, 0.75) *** | 0.31 (0.03, 0.59) * |
| **Grip strength** | 0.53 (0.33, 0.74) *** | 0.33 (-0.01, 0.68) | 0.58 (0.38, 0.78) *** | 0.31 (0.03, 0.60) * |
| **Lumbar spine** | | | | |
| **Calf circumference** | 0.41 (0.18, 0.63) *** | 0.35 (0.05, 0.64) * | 0.39 (0.17, 0.62) *** | 0.29 (0.00, 0.58) * |
| **Grip strength** | 0.36 (0.13, 0.59) ** | 0.26 (-0.12, 0.64) | 0.27 (0.03, 0.51) * | 0.02 (-0.28, 0.32) |

Unadjusted and adjusted standardized β values (95% confidence interval) for BMD associated with calf circumference or grip strength in the low- (i.e., iPTH <147.5 pg/mL) and high-iPTH (i.e., iPTH ≥147.5 pg/mL) groups are shown. Model 1 was unadjusted. Model 2 was adjusted for age, sex, history of diabetes, current smoking status, habitual alcohol drinking, hemoglobin, NRI-JH score, iPTH, and Kt/V. Exposures were included in the models separately. BMD = bone mineral density; NRI-JH = Nutritional Risk Index for Japanese Hemodialysis Patients; iPTH = intact parathyroid hormone. *$P < 0.05$; **$P < 0.01$; ***$P < 0.001$.

**Supplementary Table S4. Association between BMD and calf circumference or grip strength among hemodialysis patients based on the subgroup analysis by sex**

| | **Female group (*n* = 34)** | | **Male group (*n* = 102)** | |
|---|---|---|---|---|
| | **Model 1 (Unadjusted)** | **Model 2 (Adjusted)** | **Model 1 (Unadjusted)** | **Model 2 (Adjusted)** |
| **Femoral neck** | | | | |
| **Calf circumference** | 0.40 (0.07, 0.73) * | 0.69 (0.25, 1.13) ** | 0.49 (0.32, 0.66) *** | 0.35 (0.11, 0.58) ** |
| **Grip strength** | 0.28 (-0.06, 0.63) | 0.26 (-0.19, 0.71) | 0.40 (0.22, 0.58) *** | 0.25 (0.03, 0.47) * |
| **Lumbar spine** | | | | |
| **Calf circumference** | 0.55 (0.25, 0.85) *** | 0.52 (0.08, 0.96) * | 0.28 (0.09, 0.47) ** | 0.33 (0.08, 0.58) * |
| **Grip strength** | 0.21 (-0.15, 0.56) | 0.46 (0.07, 0.84) * | 0.07 (-0.12, 0.27) | 0.02 (-0.22, 0.25) |

Unadjusted and adjusted standardized β values (95% confidence interval) for BMD associated with calf circumference or grip strength in the female and male groups are shown. Model 1 was unadjusted. Model 2 was adjusted for age, sex, history of diabetes, current smoking status, habitual alcohol drinking, hemoglobin, NRI-JH score, iPTH, and Kt/V. Exposures were included in the models separately. BMD = bone mineral density; iPTH = intact parathyroid hormone; NRI-JH = Nutritional Risk Index for Japanese Hemodialysis Patients. *$P < 0.05$; **$P < 0.01$; ***$P < 0.001$.

**Supplementary Table S5. Correlations between bone metabolic markers and calf circumference or grip strength on the subgroup analysis using the median iPTH as a cut-off**

| | | Low-iPTH group | High-iPTH group |
|---|---|---|---|
| **Calf circumference** | **Log-transformed BALP** | −0.34 (−0.54, -0.11) ** | −0.39 (−0.57, −0.17) ** |
| | **Log-transformed P1NP** | −0.18 (−0.40, 0.07) | −0.29 (−0.49, −0.05) * |
| | **Log-transformed TRACP-5b** | −0.29 (−0.50, −0.06) * | −0.41 (−0.59, −0.19) *** |
| **Grip strength** | **Log-transformed BALP** | −0.34 (−0.54, −0.11) ** | −0.38 (−0.57, −0.16) ** |
| | **Log-transformed P1NP** | −0.13 (−0.36, 0.11) | −0.45 (−0.62, −0.24) *** |
| | **Log-transformed TRACP-5b** | −0.23 (−0.44, −0.01) | −051 (−0.67, −0.31) *** |

Correlations between log-transformed BALP, total P1NP, or TRACP-5b and calf circumference or grip strength in the low- (i.e., iPTH <147.5 pg/mL) and high-iPTH (i.e., iPTH ≥147.5 pg/mL) groups are shown. The P-values were calculated for the Pearson's product-moment correlation coefficients (r-values). BALP = bone specific alkaline phosphatase; iPTH = intact parathyroid hormone; P1NP = type I procollagen N-terminal propeptide; TRACP-5b = tartrate-resistant acid phosphatase 5b. *P < 0.05; **P < 0.01; ***P < 0.001

**Supplementary Fig. S1. Scatter plots of the relationships between bone metabolic markers and grip strength**

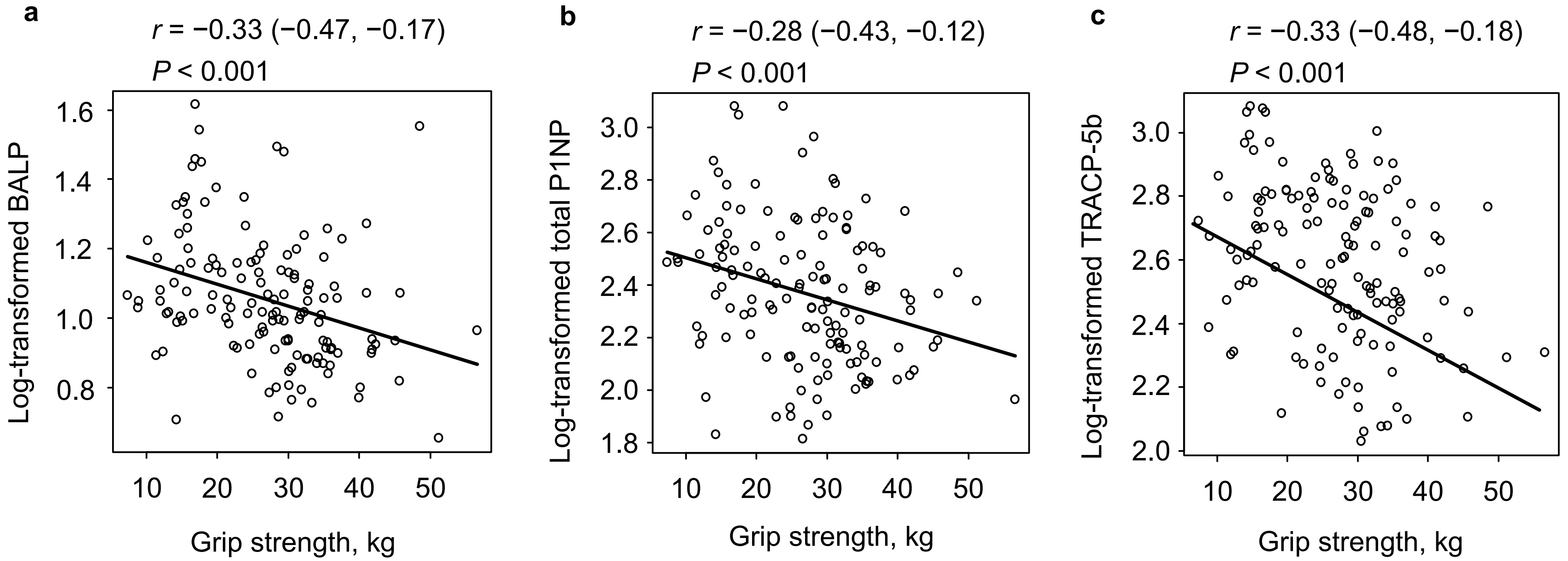


Scatter plots of the relationships between log-transformed (a) BALP, (b) total P1NP, or (c) TRACP-5b and grip strength are shown. Each circle represents an individual value. The black lines represent simple linear regression models. The *P*-values were calculated for Pearson's product-moment correlation coefficients (*r*-values). BAP = bone specific alkaline phosphatase; P1NP = type I procollagen N-terminal propeptide; TRACP-5b = tartrate-resistant acid phosphatase 5b.

**Supplementary Fig. S2. Scatter plots of the relationships between BMD and bone metabolic markers**

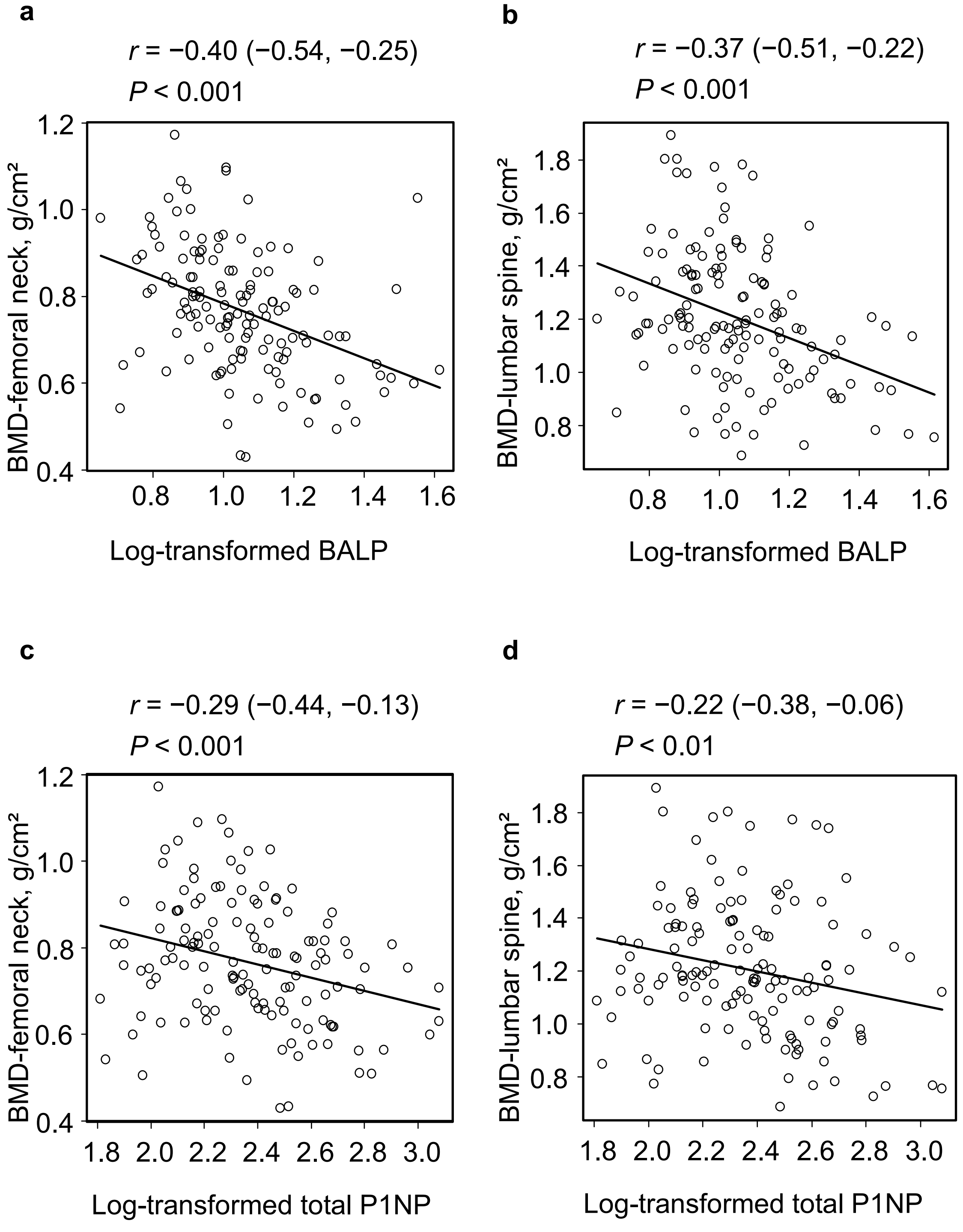

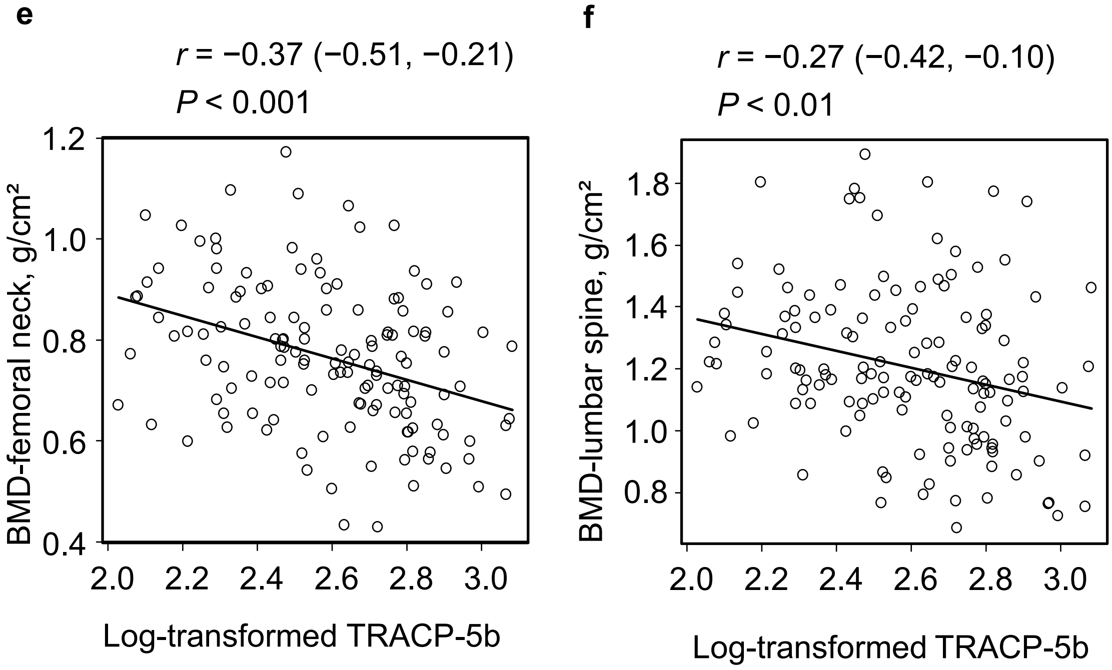


Scatter plots of the relationships between BMD at the femoral neck or lumbar spine and log-transformed (a, b) BALP, (c, d) total P1NP, or (e, f) TRACP-5b are shown. Each circle represents an individual value. The black lines represent simple linear regression models. The *P*-values were calculated for the Pearson's product-moment correlation coefficients (*r*-values). BMD = bone mineral density; BALP = bone specific alkaline phosphatase; P1NP = type I procollagen N-terminal propeptide; TRACP-5b = tartrate-resistant acid phosphatase 5b.

## Supplementary References

1. Kanda E, Kato A, Masakane I, Kanno Y. A new nutritional risk index for predicting mortality in hemodialysis patients: Nationwide cohort study. PLoS One. 2019;14(3):e0214524. doi:10.1371/journal.pone.0214524.

2. Bouillanne O, Morineau G, Dupont C, Coulombel I, Vincent JP, Nicolis I et al. Geriatric Nutritional Risk Index: a new index for evaluating at-risk elderly medical patients. Am J Clin Nutr. 2005;82(4):777-83. doi:10.1093/ajcn/82.4.777.

3. Shah B, Sucher K, Hollenbeck CB. Comparison of ideal body weight equations and published height-weight tables with body mass index tables for healthy adults in the United States. Nutr Clin Pract. 2006;21(3):312-9. doi:10.1177/0115426506021003312.

4. Daugirdas JT. Second generation logarithmic estimates of single-pool variable volume Kt/V: an analysis of error. J Am Soc Nephrol. 1993;4(5):1205-13. doi:10.1681/asn.V451205.

5. Daugirdas JT. Second generation logarithmic estimates of single-pool variable volume Kt/V: an analysis of error. J Am Soc Nephrol. 1993;4(5):1205-13.

6. Araki E, Goto A, Kondo T, Noda M, Noto H, Origasa H et al. Japanese Clinical Practice Guideline for Diabetes 2019. Diabetol Int. 2020;11(3):165-223.
